# Editorial to the Special Issue “Food Bioactives: Chemical Challenges and Bio-Opportunities”

**DOI:** 10.3390/molecules26092517

**Published:** 2021-04-26

**Authors:** Severina Pacifico, Simona Piccolella

**Affiliations:** Department of Environmental, Biological and Pharmaceutical Sciences and Technologies, University of Campania “Luigi Vanvitelli”, Via Vivaldi 43, 81100 Caserta, Italy; simona.piccolella@unicampania.it

This Special Issue, entitled “Food Bioactives: Chemical Challenges and Bio-Opportunities”, was born with the aim of attracting contributions on analytical challenges in food bioactives’ chemistry and bioactivity, which form the basis of proper bio-opportunities. To date, it represents a collection of nine original research papers and one review article that extended scientific knowledge regarding the chemical constituents of edible plant extracts and their role as health-promoting agents. Innovation and originality in the research scope, design of experiments and results were highlighted in all the subtopics covered by the enclosed articles, which included medicinal and aromatic plant (MAP) ingredients, ancient foods nowadays re-valued for their functionality, compounds derived from food processing procedures, analytical methods related to food quality and agro-wastes as innovative and renewable sources for nutraceuticals.

MAPs are well-known species widely used in several sectors, e.g., pharmaceutical, cosmetic, liquor and food industries, due to their richness in biologically active ingredients, which can be classified based on their chemical features and/or therapeutic effects [1]. *Aloe vera* is one of the most used plants with a great commercial spread due to the multiple health-beneficial effects exerted by its pulp or gel constituents. The awareness that the soluble and fermentable fiber components of *Aloe* possess prebiotic effects able to modulate the composition of the microbiota, making them useful in the food industry, led Tornero-Martínez et al. to obtain new insights into the changes in the fiber and free phenols during in vitro digestion and colon fermentation of aloe gel and polysaccharide extract [2]. Their results showed that the behavior of the latter was similar to that of lactulose, suggesting the possible use as a prebiotic.

A novel perspective in the exploitation of MAPs was represented by the paper of Ielciu and co-workers, in which fresh young shoots of *Rosmarinus officinalis* L. were macerated in 90% ethanol to obtain a tincture, which was tested against liver tissue damages [3]. The phytochemical analysis led to the identification and quantification of polyphenols and terpenes by HPLC–UV–MS and GC–MS, respectively. The authors highlighted that this rosemary organ, besides the most studied leaves, could also represent a valuable plant material for the formulation of products with hepatoprotective properties through antioxidant mechanisms, which they investigated in rats with experimentally induced hepatotoxicity.

In addition to MAP-derived extracts, pure thymol and carvacrol, two phenolic terpenoids mainly derived from essential oils of thyme and oregano, were investigated for their antibacterial activity against *Staphylococcus aureus* [4]. Both proved to be promising antibacterial agents, which could likely be involved in counteracting bacterial resistance to antibiotics.

A full awareness of the role played by a healthy diet, as part of a healthy lifestyle, in countering or slowing down chronic and degenerative diseases has strongly increased the interest in food bioactives and the return of ancient but nowadays considered functional foods. This is the case of hemp seeds, which represented a significant source of nutrition for thousands of years and have recently been re-evaluated from a nutraceutical perspective [5]. In this context, Nigro et al. deeply investigated the chemical diversity of hemp seed phenylpropanoid amides and their derivative lignanamides by means of high-resolution tandem mass spectrometry (HR-MS/MS) techniques. They highlighted that these molecules were able to inhibit U-87 glioblastoma cell line survival and migration, inducing apoptosis while suppressing autophagy, whereas they interestingly did not exert cytotoxicity in nontumorigenic human fibroblasts [6].

The investigation of food metabolic composition is very challenging, as it is a very complex biological matrix. Thus, proper approaches that employ chromatographic separation coupled with UV and mass spectrometry detection, as described in the above-mentioned paper, are required. Furthermore, there is an urgent need to make the methods as sustainable as possible, e.g., by replacing chlorinated solvents in the extraction protocols of less polar compounds. In the light of the above, Cortés-Herrera et al. developed a greener alternative to the recovery and simultaneous analysis of carotenoids and fat-soluble vitamins from Costa Rican avocados, fruits of high economic value and of dietary interest, especially in Latin American cuisine [7]. Analytical methods, which are becoming increasingly powerful, can also enhance our knowledge about food quality indices; when jointly used with chemometric tools, they allowed discriminating between different *Pistacia vera* oils according to their quality profiles [8]. The proposed methodology had a number of advantages, including being accurate and not time- or solvent-consuming, and proved to be promising in fraud detection related to plant-derived oils.

The role that dietary substances, to which nutraceutical attributes are increasingly entrusted, play in halting or reversing oxidative stress-related diseases has been ever more deeply investigated, especially in relation to cancer [9]. The review article enclosed in the present paper collection focused its attention on cytotoxic, antitumor and antimetastatic properties of *Urtica dioica* L. extracts; it was based on previous investigations employing both human cancer cell lines and in vivo models, with particular attention devoted to breast cancer [10]. The authors concluded that thanks to its richness in bioactive compounds, *U. dioica* could be used as a promising source of nutraceuticals useful as chemopreventive and/or chemoprotective agents in cancer disease onset and development. Breast cancer is considered the most prevalent cancer among women and one of the main causes of death worldwide. It was also the focus of another research article in this Special Issue, which dealt with a promising cytotoxic pistachio hull ethyl acetate extract that could be considered in the future as part of anticancer drug treatment [11].

It is worth noting that plant sources for nutraceutical compounds are not limited to the edible organs of the plant itself; interestingly, such compounds can also be recovered from by-products and wastes, giving them new life in the prevention of human diseases. Piccolella et al. took into consideration wine production wastes, focusing in particular on the leaves of a *Vitis vinifera* cultivar named “Greco di Tufo”, which represents a great resource of the Campania Region (Italy) territory, as it received the quality classification “Controlled and Guaranteed Designation of Origin” (DOCG acronym in Italy) [12]. The authors applied an extraction/fractionation protocol that favored the obtainment of a mixture of flavonol glycosides and glycuronides variously oxygenated at the B-ring. The isolation of the most abundant compound led to its full chemical characterization by spectroscopic and HR-MS/MS tools. Then, the absence of cytotoxicity in preliminary tests using central nervous system cell lines paved the way for further exploitation of their neuroprotective potential.

Bioactives’ presence and abundance are strictly related to their food source. However, food could contain other constituents, beyond naturally occurring compounds, whose presence is processing-induced. For example, during deep-frying processes, vegetal oils become a source of lipid peroxidation products due to the exposure to high temperatures. The study of Zaunschirm et al. aimed at investigating the impact of linoleic acid and its primary and secondary peroxidation products on genomic and metabolomic pathways in human gastric cells (HGT-1). They demonstrated that these substances were able to influence the pathways related to amino acid metabolism, thus affecting metabolic health [13].

In conclusion, the contributions in this Special Issue underscore the potential of exploiting plants, beyond nutrition, as “biofactories” of compounds useful for human health and well-being.
